# Unexpected discrepancies in hospital administrative databases can impact the accuracy of monitoring thyroid surgery outcomes in France

**DOI:** 10.1371/journal.pone.0208416

**Published:** 2018-12-06

**Authors:** Frederic Mercier, Nathalie Laplace, Elliot J. Mitmaker, Cyrille Colin, Jean-Louis Kraimps, Frederic Sebag, Stephanie Bourdy, Antoine Duclos, Jean-Christophe Lifante, On Behalf of The CATHY Study Group

**Affiliations:** 1 Department of General and Endocrine Surgery, Lyon Sud Hospital, Hospices Civils de Lyon, Pierre-Bénite, France; 2 Surgical oncology department, Centre hospitalier universitaire de Montreal, Montreal, Qc, Canada; 3 Department of Surgery, McGill University, Montreal, Qc, Canada; 4 Pôle Information Médicale Evaluation Recherche Hospices Civils de Lyon, Lyon, France; 5 Department of Endocrine Surgery, Poitiers University, Jean Bernard Hospital, Poitiers, France; 6 Assistance Publique-Hôpitaux de Marseille, La Timone Hospital, Marseille, France; 7 Center for Surgery and Public Health, Brigham and Women's Hospital—Harvard Medical School, Boston, MA, USA; 8 EA 7425 HESPER, Health Services and Performance Research, University Claude Bernard Lyon 1, Domaine Rockefeller, Lyon, France; Universidade de Mogi das Cruzes, BRAZIL

## Abstract

**Objective:**

To determine the validity of hospital administrative databases compared to prospective collection of medical data assessing thyroid surgery complications.

**Background:**

Administrative data are increasingly used to track surgical outcomes.

**Methods:**

All patients undergoing thyroid surgery at three French university hospitals between April 2008 and April 2009 were prospectively included. Using diagnosis and procedural codes from hospital administrative database, we designed three indicators for measuring complications of thyroid surgery: recurrent laryngeal nerve palsy, postoperative hypoparathyroidism, and postoperative hemorrhage. Gold standard was obtained from a prospective collection of medical data after systematically screening each patient for the above-mentioned complications. Their ability to monitor surgical outcomes over time within individual hospitals was estimated using control charts. Spatial comparison between hospitals was performed by funnel plots.

**Results:**

A total of 1909 patients were included. Complication rates extracted from administrative data were significantly lower compared to medical data (nerve palsy 2.4% vs. 6.7%, hypoparathyroidism 10.6% vs. 22.3%, p<0.0001). Indicator sensitivity was 30.4% for nerve palsy, 45.4% for hypoparathyroidism and 71.4% for postoperative hemorrhage. Corresponding positive predictive values were 84.4%, 95.1% and 68.2%. In two of the three hospitals, administrative data were not able to track temporal variations in complications rates. Regarding inter-hospital comparisons, 2 out of 3 hospitals were considered outliers according to administrative data despite having an average performance based on medical data.

**Conclusions:**

The ability of indicators extracted from administrative databases to measure thyroid surgery outcomes depends on the quality of underlying data coding. Validation in every center should be a prerequisite before implementing such metrics for tracking performance

## Introduction

Hospital administrative data are increasingly used for assessing surgical outcomes. Because of its low cost and ease of availability, this source of patient information is considered to be credible when reviewing medical records and reporting system errors for tracking surgeon performance [1–4]. However, the underlying data collection is strongly influenced by economic issues, as data coders are mostly instructed to invoice patient hospitalization rather than accurately reporting adverse events. This mechanism of data recording may limit the validity of analyzing complication rates across institutions or conducting epidemiological studies. To avoid this pitfall, indicators must be carefully extracted from administrative databases and validated prior to use. For instance, patient safety indicators (e.g., postoperative sepsis or pulmonary embolism) were developed and tested according to a rigorous validation process, with the goal of reporting the complication rate within each individual hospital [5,6].

There are three major complications attributable to thyroid surgery in the postoperative setting that can impact a patient’s daily activities and even be life-threatening: recurrent laryngeal nerve palsy, hypoparathyroidism and hemorrhage [7]. The prevalence of recurrent laryngeal nerve palsy ranges from 2.0% to 7.5% based on prospectively collected medical data [7–10] versus 0.5% to 0.8% [11–13] from administrative data sources. Similarly, hypoparathyroidism following thyroidectomy ranges from 7.3% to 25.2% using medical data [7,10,14] versus 0.3% to 6.2% according to administrative data. [11–13] Finally, postoperative hemorrhage requiring reoperation occurs in 0.7% to 1.7% of cases [7,15].

The purpose of this multicenter study was to develop and assess the validity of novel patient safety indicators in thyroid surgery. In particular, each indicator was extracted from administrative databases and compared against the gold-standard of a prospectively collected medical database to estimate the indicator criterion validity and its potential for tracking surgical outcomes within and between hospitals.

## Methods

### Study design and indicators

This was a validation study investigating the following question: Does the indicator developed from administrative data distinguish patients with and without the target complication among patients who underwent thyroid surgery [16]. All patients undergoing thyroid surgery in three French academic hospitals (Lyon, Marseille and Poitiers) from April 2008 to April 2009 were included. The Research Committee for the Protection of Persons allowed the study in accordance with ethical directives. The National Advisory Committee on Information Processing in Material Research in the Field of Health also approved the study, regarding the anonymous processing of personal health information. The participating centers approved the study protocol without giving incentives to surgeons for their participation. The ethics committee waived the requirement for patient consent. Before surgery, patients received written information about personal data use, and gave verbal consent for sharing their data.

The screening of postoperative complications was performed by running extraction algorithms through hospital administrative data. These data included information about all inpatient hospitalizations that occurred in participating institutions. Standard discharge summaries for each hospitalization contained compulsory information about the patient (e.g., gender and age), primary and secondary diagnoses using the Tenth International Classification of Diseases (ICD-10), as well as the associated procedural codes. In France, large national databases are used for reimbursement and have a coding system with strict definitions, with a subset of records undergoing regular audits so as to measure the rate of coding error.

Three indicators were designed to measure major postoperative complications in thyroid surgery: recurrent laryngeal nerve palsy, postoperative hypoparathyroidism and postoperative hemorrhage. Each indicator was calculated as a ratio between the complication of interest and the population at risk. The denominator for calculating recurrent laryngeal nerve palsy and postoperative hemorrhage risk included all patient who underwent thyroid surgery (S1 and S2 Figs), while patients who only underwent total thyroidectomy were selected for hypoparathyroidism since no risk of hypoparathyroidism exists in cases of hemithyroidectomy (S3 Fig). To determine recurrent laryngeal nerve palsy and postoperative hypoparathyroidism numerators, hospital records with ICD-10 codes J38.0 (“Paralysis of vocal cords and larynx”) and E89.2 (“Postprocedural hypoparathyroidism”) were considered. Regarding postoperative hemorrhage, the numerator included the presence of either procedural codes “Reoperation for bleeding control, by cervicotomy” or “Reoperation for evacuation of deep cervical space, by cervicotomy”. (S1 Fig)

### Standard medical chart reviewing

Each potential complication was then compared in a blinded fashion with information obtained from a prospectively collected medical database using a rigorous screening protocol to detect complications for every patient in the database. Postoperative outcomes were systematically assessed during hospitalization within 48 hours after thyroid surgery. Postoperative vocal cord mobility was assessed by laryngoscopy for each patient. Serum calcium concentration was measured only for patients who underwent a total thyroidectomy. Postoperative hypoparathyroidism was defined as a serum calcium concentration below 2 mmol/l or a requirement for vitamin D and/or calcium supplementation to maintain calcium concentrations within normal limits following thyroidectomy. Furthermore, a patient report form was completed after discharge which included items regarding procedure performed, surgical indication and related complications based on information gathered from the medical record.

### Statistical validation

Criterion validity was assessed using administrative algorithms and comparing them to the prospectively collected medical database from each surgeon or department. Each potential complication was flagged in the administrative database and compared with medical data in order to be categorized as either a true or false positive. Undetected complications in the administrative database were classified as a true or false negative depending on the presence or absence of a complication found within our database. Sensitivity, specificity, and positive and negative predictive values of each indicator were then determined with a corresponding 95% confidence interval (CI95%).

Secondly, the correlation between administrative and medical data to track complication rates over time was computed for each indicator on a monthly basis, for each hospital, using the Spearman’s rank correlation coefficient. A *ρ* coefficient *≥ 0*.7 was considered as a strong correlation [17,18]. When a strong correlation was found, two Shewhart control charts were then generated using administrative and medical data, respectively. Combining time series analysis with graphical presentation of data, each data point on the control charts represented the observed incidence of complications per month for a given hospital [19,20]. Control and warning limits were respectively set at two and three standard deviations of the mean proportion of complications [21]. Agreement in signal detection between the two control charts was estimated using weighted kappa statistics. Satisfactory agreement was obtained in case of κ values greater than 0.70 [22].

Third, the ability of administrative data to discriminate hospitals according to cross-sectional comparison of their respective complication rates was established using funnel plots. The funnel plot displays the performance of each institution on the same graph, based on their respective volumes of activity and overall complication rate during the study period. Limits were determined in order to categorize each hospital as a very high performer (<3Standard Deviations from the mean), high performer (from -3 to -2 SD), average performer (from -2 to +2 SD), poor performer (from +2 to +3 SD) and very poor performer (>3SD).

All analyses were conducted using SAS^®^ 9.2 (SAS Institute, Cary, North Carolina, USA) and statistical significance for analyses was set at p<0.05.

## Results

**Fig 1:**
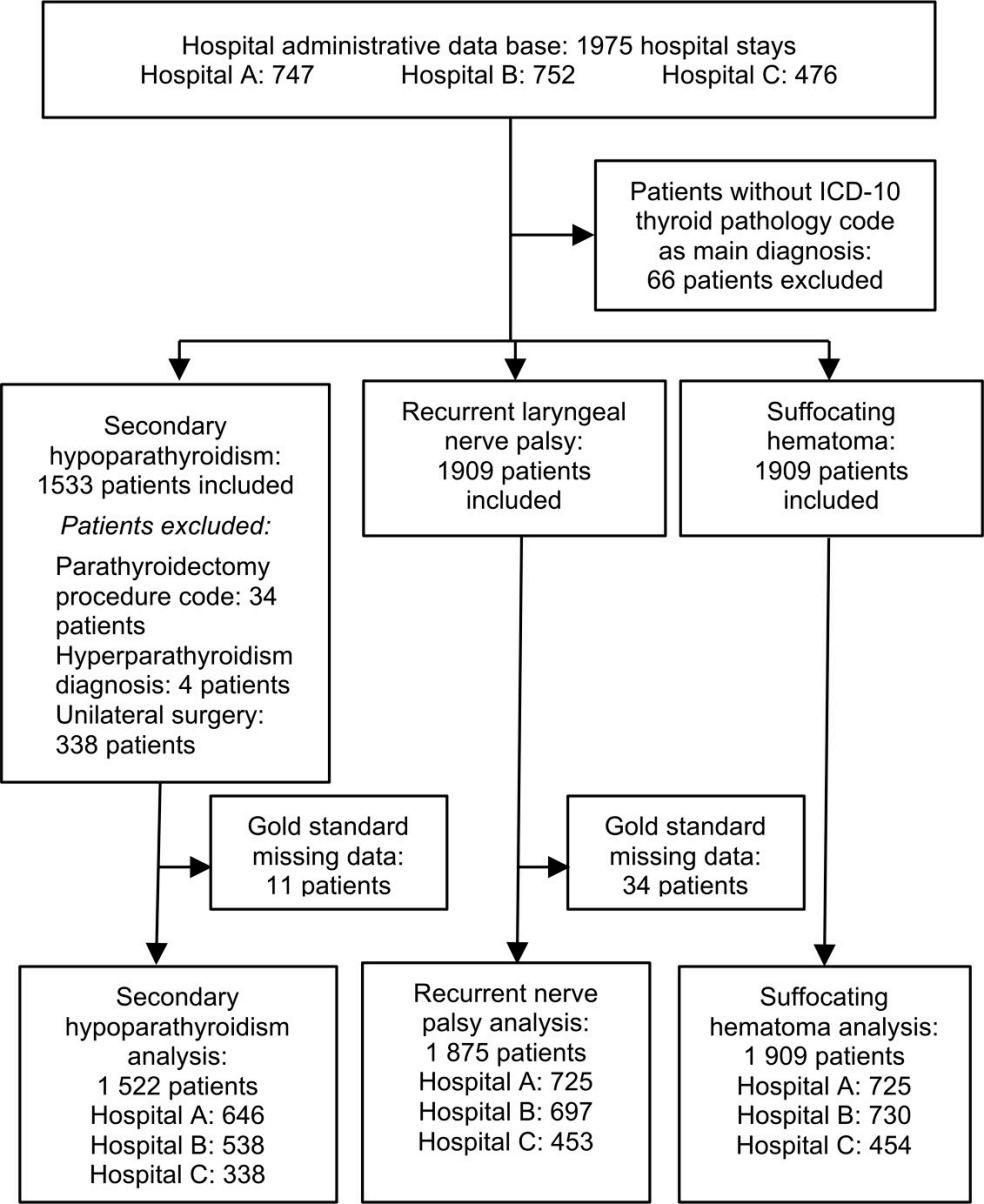
Study flowchart.

A total of 1,975 patients underwent a thyroid procedure during the study period, 66 were excluded because the main diagnostic ICD codes were not related to thyroid pathology. Patients clinical characteristics are described in Table 1. As shown in Fig 1, the final sample included 1875 patients for recurrent laryngeal nerve palsy (exhaustivity of 98.2%), 1522 patients for hypocalcemia (99.3%) and 1909 patients for postoperative hemorrhage (100%). There were substantial discrepancies between data sources relative to the coding of diagnosis and procedural codes. Thyroid carcinoma was particularly overrepresented in administrative data compared to medical data (20.1% vs 11.5%, p<0.001).

**Table 1:** Population characteristics and procedures performed.

	**Hospital administrative database**	**Medical data**				***P***
**N**		1 909		1 909		
**Age (years)**		51,4 ± 15,1		51,4 ± 15,1		0,5
**Sex (M/F)**		430 / 1479		430 / 1479		0,6
**Diagnosis**						
	Multinodular goiter or thyroid nodules	1 230	(64,4%)	1 305	(68,4%)	<0,001
	Graves disease	175	(9,2%)	187	(9,8%)	0,07
	Other hyperthyroidism	107	(5,6%)	170	(8,9%)	<0,001
	Thyroid carcinoma	384	(20,1%)	220	(11,5%)	<0,001
	Other	13	(0,7%)	26	(1,4%)	<0,05
**Main procedure**						
	Hemithyroidectomy	341	(17,9%)	333	(17,4%)	0,3
	Total thyroidectomy	1 505	(78,8%)	1 449	(75,9%)	<0,001
	Subtotal thyroidectomy	6	(0,3%)	18	(0,9%)	<0,01
	Reoperation	47	(2,5%)	100	(5,2%)	<0,001
	Other	10	(0,5%)	9	(0,5%)	1,0
**Lymph node dissection**						
	Total	131	(6,9%)	144	(7,5%)	<0,05
	Ipsilateral central compartment	41	(2,1%)	52	(2,7%)	0,07
	Bilateral central compartment	45	(2,4%)	45	(2,4%)	1,0
	Lateral	34	(1,8%)	10	(0,5%)	<0,001
	Central + lateral	10	(0,5%)	35	(1,8%)	<0,001
	Other	1	(0,1%)	2	(0,1%)	1,0

Rates of postoperative recurrent laryngeal nerve palsy estimated from administrative (2.4%, CI95% 1.4%-3.1%) and medical data (6.7%, CI95% 5.5%-7.8%), were significantly different (Table 2, p<0.001). Among the 45 patients flagged in the administrative database, 38 were true positives and 7 were false positives, while 87 patients with postoperative nerve palsy were not flagged (false negatives). Overall, indicator for sensitivity and PPV were 30.4% (95%CI, 22.3%-38.5%) and 84.4% (95%CI, 73.9%-95.0%), respectively. The sensitivity ranged from 4.8% for Hospital C to 70.8% for Hospital A.

**Table 2:** Criterion validity of the indicators of thyroid surgery from Hospital administrative database versus Medical data.

	TP	FP	TN	FN	Sensitivity (%) (CI 95%)		Specificity (%) (CI 95%)		PPV (%) (CI 95%)		NPV (%) (CI 95%)		Hospital administrative database complication rate (%) (CI 95%)		Medical data complication rate (%) (CI 95%)	
**Indicator**																
**Recurrent laryngeal nerve palsy**																
Hospital A	34	5	672	14	70,8	(58,0–83,7)	99,3	(98,6–99,9)	87,2	(76,7–97,7)	98,0	(96,9–99,0)	5,4	(3,7–7,0)	6,6	(4,8–8,4)
Hospital B	2	2	660	33	5,7	(0–13,4)	99,7	(99,3–100)	50,0	(1,0–99,0)	95,2	(93,7–96,8)	0,6	(0,0–1,1)	5,0	(3,4–6,6)
Hospital C	2	0	411	40	4,8	(0–11,2)	100	(100–100)	100	(100–100)	91,1	(88,5–93,8)	0,4	(0,0–1,1)	9,3	(6,6–11,9)
Total	38	7	1 743	87	30,4	(22,3–38,5)	99,6	(99,3–99,9)	84,4	(73,9–95,0)	95,2	(94,3–96,2)	2,4	(1,7–3,1)	6,7	(5,5–7,8)
**Postoperative hypoparathyroidism**																
Hospital A	97	1	518	30	76,4	(69,0–83,8)	99,8	(99,4–100)	99,0	(97,0–100)	94,5	(92,6–96,4)	15,2	(12,4–17,9)	19,7	(16,6–22,7)
Hospital B	1	0	411	126	0,8	(0–2,3)	100	(100–100)	100	(100–100)	76,5	(73,0–80,1)	0,2	(0,0–0,6)	23,6	(20,0–27,2)
Hospital C	56	7	246	29	65,9	(55,8–76,0)	97,2	(95,2–99,3)	88,9	(81,1–96,7)	89,5	(85,8–93,1)	18,6	(14,5–22,8)	25,1	(20,5–29,8)
Total	154	8	1 175	185	45,4	(40,1–50,7)	99,3	(98,9–99,8)	95,1	(91,7–98,4)	86,4	(84,6–88,2)	10,6	(9,1–12,2)	22,3	(20,2–24,4)
**Postoperative hemorrhage**																
Hospital A	2	0	719	4	33,3	(0–71,1)	100	(100–100)	100	(100–100)	99,5	(98,9–100)	0,3	(0,0–0,7)	0,8	(0,2–1,5)
Hospital B	5	6	717	2	71,4	(20,5–93,8)	99,2	(98,7–99,9)	45,5	(15,1–73,8)	99,7	(99,1–100)	1,5	(0,6–2,4)	1,0	(0,3–1,7)
Hospital C	8	1	445	0	100	(100–100)	99,8	(99,3–100)	88,9	(68,4–100)	100	(100–100)	2,0	(0,7–3,3)	1,8	(0,6–3,0)
Total	15	7	1 881	6	71,4	(52,1–90,8)	99,6	(99,4–99,9)	68,2	(48,7–87,6)	99,7	(99,4–99,9)	1,2	(0,7–1,6)	1,1	(0,6–1,6)

Rates of postoperative hypoparathyroidism estimated from administrative (10.6%, CI95% 9.1%-12.2%) and medical data (22.3%, CI95% 20.2%-24.4%), were also significantly different (Table 2, p<0.001). Among the 162 patients flagged in administrative database, 154 were true positives and 8 were false positives, while 185 patients with postoperative hypoparathyroidism were not flagged. Overall, indicator for sensitivity and PPV were 45.4% (95%CI, 40.1%-50.7%) and 95.1% (95%CI, 91.7%-98.4%), respectively. The sensitivity ranged from 0.8% for Hospital B to 76.4% for Hospital A.

Rates of postoperative hemorrhage estimated from administrative (1.2%, CI95% 0.7%-1.6%) and medical data (1.1%, CI95% 0.6%-1.6%), were not different. Among the 22 patients flagged in the administrative database, 15 were true positives and 7 were false positives, while 8 patients with postoperative hematoma were not flagged (false negatives). Overall, indicator for sensitivity and PPV were 71.4% (95%CI, 52.1%-90.8%) and 68.2% (95%CI, 95%CI, 48.7%-87.6%), respectively. The sensitivity ranged from 33.3% for Hospital A to 100% for Hospital C.

**Fig 2:**
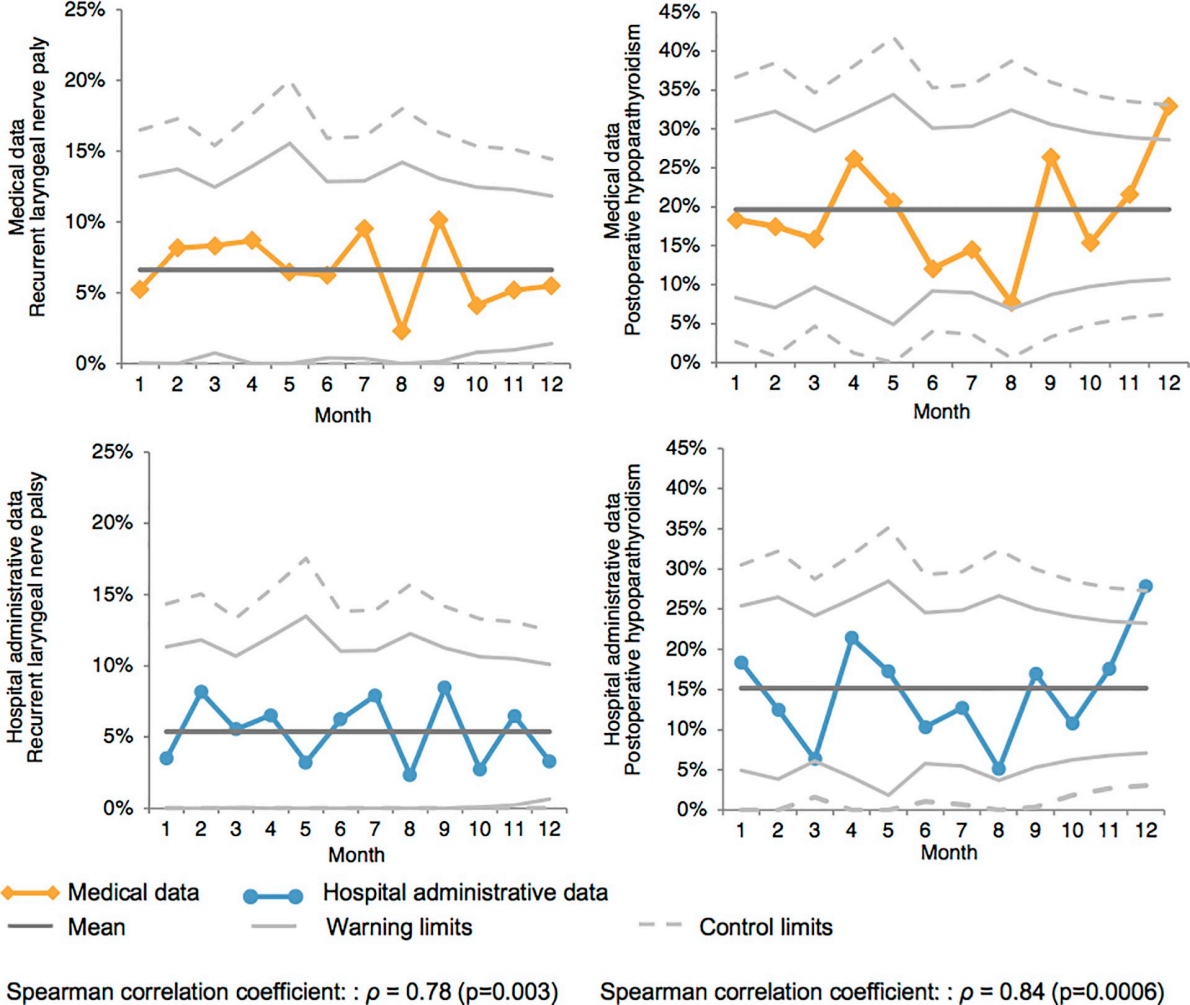
Monthly report control chart in hospital A. 2a: control chart reporting monthly proportions of recurrent laryngeal nerve palsy 2b: control chart reporting monthly proportions of postoperative hypoparathyroidism.

In contrary to other hospitals, a strong correlation was observed in Hospital A between administrative and medical data to track monthly rates of recurrent nerve palsy (*ρ* = 0.78, p<0.01) and hypoparathyroidism (*ρ* = 0.84, p<0.001) (Table 3). Corresponding control charts are presented in Fig 2, revealing excellent agreement between data sources for hospital A (recurrent nerve palsy *κ* = 0.78 and hypoparathyroidism *κ* = 0.80). The only single point outside the upper limits at the twelfth month for hypoparathyroidism was detected by control charts based both on administrative and medical data. There was no other point beyond the limits on either of the control charts.

**Table 3:** Agreement between Hospital administrative database and medical data.

	Recurrent laryngeal nerve palsy *κ* values (CI 95%)			Postoperative hypoparathyroidism *κ* values (CI 95%)	
Hospital					
A		0,8	(0,4–1)	0,8	(0,5–1)
B		0,1	(-0,4–0,5)	0,1	(-0,2–0,2)
C		0,1	(-0,2–0,2)	0,2	(-0,1–0,5)
All hospitals		0	(-0,2–0,2)	0,2	(0,02–0,4)

Interpretation of *κ values*^*30*^: <0,4: poor agreement; 0,4–0,75: moderate agreement; >0,75: excellent agreement

**Fig 3:**
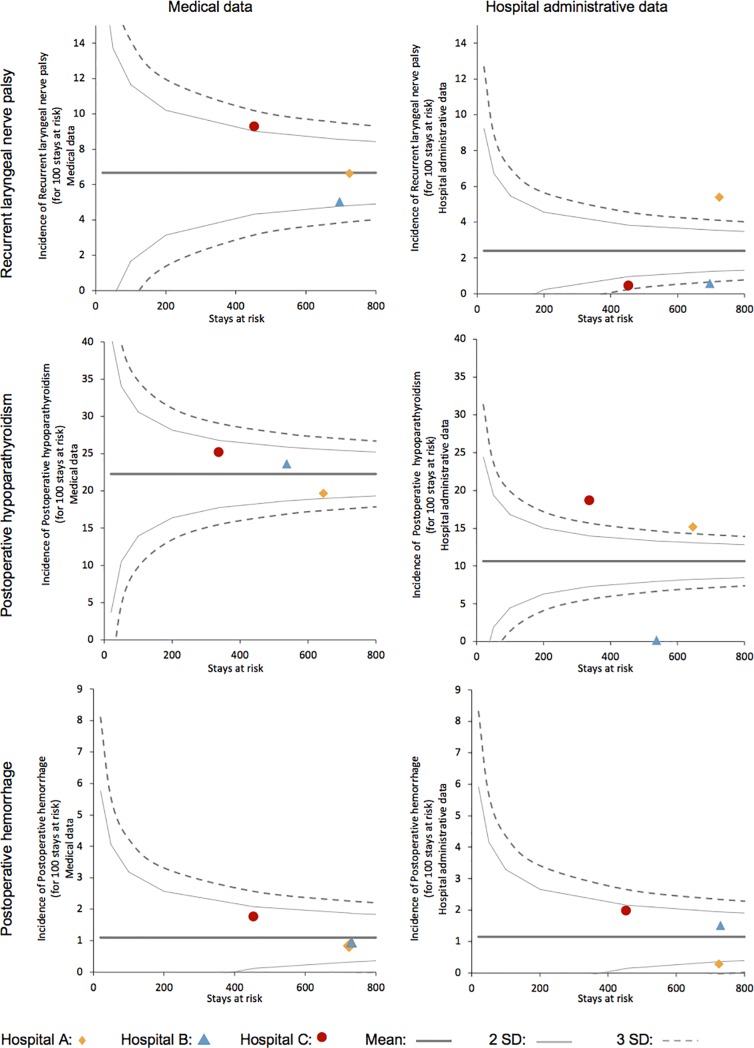
Funnel-plot of complication rates: Hospital administrative data versus medical data.

Fig 3 reveals important variations of the performance of all hospitals if the analysis is done with medical chart or hospital administrative data. In example the hospital C is a poor performer when analyzed with the medical chart but high performer when weighed by hospital administrative data.

## Discussion

Data collected from hospital information systems are increasingly employed for research purpose or surgical performance assessment. Critical metrics extracted from these administrative databases have not been previously validated to ensure that they accurately match direct medical observation. Yet using invalid indicators can undoubtedly lead to erroneous conclusions. In this study, we analyzed the validity of administrative data to report thyroid surgery outcomes. Additionally, we considered their value for monitoring surgical teams’ performance over time within individual hospitals or performing inter-hospital comparisons.

A prerequisite before implementing patient safety indicators relies on their sensitivity and positive predictive value, meaning their ability to detect complications without false positives. Our results suggest a poor sensitivity regarding two indicators of major adverse events following thyroid surgery: “recurrent laryngeal nerve palsy” and “post-operative hypoparathyroidism”. Modest ability of these indicators to detect complications has to be tempered by the heterogeneity in data coding between hospitals. Monitoring of surgical outcomes revealed variability in 2 out of 3 hospitals, while only one institution demonstrated a high correlation between administrative and medical data. Since the 3 hospitals work on the same system it can be safely assumed that the difference in correlation performance from the third hospital is related to an individual coder performance. Furthermore, benchmarking of hospital performance based on administrative data was inappropriate due to erroneous coding of complications across institutions. As a result, average performing hospitals were falsely detected as a poor performing or top performing hospital, depending on their transparency or lack thereof in coding postoperative complications accurately. According to Donabedian [23], these indicators depend on four factors: chance, case mix, data collection, and quality. The first three factors must be controlled before attempting to perform an inter-hospital quality comparison. The first two factors (i.e. chance and case-mix) appear controlled by the large number of cases in each hospital (more than 700 in 2 hospitals and approximately 500 in the remaining hospital) But data collection is not similar in each hospital, therefore invalidating the quality comparison factor.

Imperfect coding of diagnoses and procedures performed is common in hospital information systems [24]. Although no study has assessed indicator validity for measuring specific complications following thyroid surgery, our estimates are consistent with several studies incriminating the validity for most patient safety indicators developed from administrative databases by the American Agency for Healthcare Research and Quality (AHRQ-PSI) [25,26]. Rosen et al [27] have explored their validity in comparison to medical records of the Veterans Health Administration, while Romano et al [28] have selected the American College of Surgeons National Surgical Quality Improvement Program as the gold standard (ACS-NSQIP). They both reported major disagreements related to inaccuracy or heterogeneity in coding practices across institutions, which are partly explained by the lack of “present on admission” information or meaningful codes.

The higher sensitivity of the indicator for postoperative hemorrhage probably derives from the fact that administrative databases are based on procedural rather than diagnostic codes. All procedures that a patient undergoes during their hospital stay are systematically coded by surgeons at the time of surgery, while diagnosis related to postoperative complications may be forgotten because they are usually collected at discharge. Coding can be heavily influenced by economic issues, which may explain the observed differences between data sources regarding accuracy of principal diagnosis (like thyroid carcinoma). Additionally, in the absence of a "present on admission" qualifier, it is difficult to distinguish postoperative complications that occurred during a hospital stay from patient comorbidities.

The major strength of our study relates to its multicentric prospective design, and high inclusion rate in the predetermined period. The occurrence of postoperative complication was systematically assessed and coded in administrative databases in a blinded fashion. Conversely, our prospectively collected medical databases relied on a rigorous and homogeneous protocol to detect complications amongst participating centers. However, we have to acknowledge several limitations of this study. The coding of routine data may be driven by financial incentives both at the individual and collective levels. Generalizing the results to every hospital is questionable, as our study sample was focused on academic centers performing high volumes of thyroidectomies. Furthermore, the hospitals’ public status as well as demographic characteristics varied and may have accounted for differences in data coding. Moreover, our study was conducted over a single year, which does not allow consideration of the potential impact of billing changes on data coding over longer periods. Finally, our study concerned only thyroid surgery and therefore may not be applicable to other surgical areas.

This validation study raises important concerns about the utilization of administrative data for investigating surgical outcomes. Due to their high availability and low cost, these data sources are increasingly employed by researchers and health administrators alike. However, indicators extracted from these databases to track surgical complications need to be validated before utilizing the information to make important hospital administrative decisions. Ironically, hospitals with transparent coding practices run the risk of being labelled as an under-performer, while those omitting their complications may falsely be considered as top performers. In light of these findings, exploiting administrative data for comparing hospital performance should be rejected until a more appropriate measure of validation can be achieved. In the meantime, surgical teams using reliable coding practices can benefit from monitoring their outcomes and mitigate any potential errors from being inadvertently published.

## Supporting information

S1 Fig Description of the indicator recurrent laryngeal nerve palsy. (DOCX)

S2 Fig Description of the indicator reoperation for bleeding control. (DOCX)

S3 Fig Description of the indicator postoperative hypoparathyroidism. (DOCX)
