# Supplementary material for: Unexpected discrepancies in hospital administrative databases can impact the accuracy of monitoring thyroid surgery outcomes in France
Source: PLoS One. 2018 Dec 6;13(12):e0208416. doi: 10.1371/journal.pone.0208416 (PMC6283582; doi:10.1371/journal.pone.0208416)
Supplement: S1 Fig — (DOCX) [file pone.0208416.s001.docx]

| **S1 Figure.**  Description of the indicator «Recurrent laryngeal nerve palsy» | |
| --- | --- |
| Definition | The indicator «Recurrent laryngeal nerve palsy» allows measuring the rate of recurrent laryngeal nerve palsy after a thyroid surgery, for an institution. |
| Importance | The recurrent laryngeal nerve palsy is one of the two potentially preventable adverse events after thyroid surgery. An accurate indicator for this complication could make possible for teams to focus their potential to improve their performance, in order to reduce the occurrence of this complication over time. |
| Type of indicator | Outcome indicator |
| Collection | Continuously collection |
| Data sources | - Hospital admministrative database « Programme de Médicalisation des Systèmes d’Information en Médecine » - Medical record data collection |
| Nature of the indicator | Number of thyroid surgery patients per 100 with a recurrent laryngeal nerve palsy (rate) |
| Method of calculation | Patients with a ICD-10 diagnosis code of recurrent laryngeal nerve palsy  = x 100  Inpatient stays with a thyroid procedure code |
| Numerator | Among the patients in the denominator, patients with one of the following ICD-10 diagnosis codes:   - J38.0: Paralysis of vocal cords and larynx   * ICD-10 codes not retained in the final numerator |
| Denominator | Inclusion criteria:   - Includes inpatients with a thyroid procedure code among: - KCFA004 : Isthmus resection, by cervicotomy - KCFA001 : Lobectomy, by cervicotomy - KCFA008 : Lobectomy and isthmus resection, by cervicotomy - KCFA010 : Subtotal thyroidectomy, by cervicotomy - KCFA005 : Total thyroidectomy, by cervicotomy - KCFA001 : Totalisation of thyroidectomy, by cervicotomy - KCGA001 : Thyroid nodule resection, by cervicotomy - KCFA009 : Subtotal thyroidectomy, by cervico-thoracotomy - KCFA007 : Total thyroidectomy, by cervico-thoracotomy - KCFA002 : Total thyroidectomy associated with a laryngeal cartilage resection, by cervicotomy - KCFA003 : Total thyroidectomy associated with a non-interrupted tracheal resection and anastomosis, by cervicotomy - KCFA006 : Total thyroidectomy associated with a interrupted tracheal resection and anastomosis, by cervicotomy   Exclusion criteria:   - Excludes patients without ICD-10 thyroid pathology code as main diagnosis, among: - E04.2: Nontoxic multinodular goitre - E04.1: Nontoxic single thyroid nodule - E04.8: Other specified nontoxic goitre - E04.9: Nontoxic goiter, unspecified - E05.0: Thyrotoxicosis with diffuse goitre - E05.1: Thyrotoxicosis with toxic single thyroid nodule - E05.2: Thyrotoxicosis with toxic multinodular goitre - E05.3: Thyrotoxicosis from ectopic thyroid tissue - E05.8: Other thyrotoxicosis - E05.9: Thyrotoxicosis, unspecified - C73: Malignant neoplasm of thyroid gland - D34: Benign neoplasm of thyroid gland - D44.0: Neoplasm of uncertain or unknown behavior of endocrine gland – thyroid gland - E06.3: Autoimmune thyroiditis - E06.5: Other chronic thyroiditis - E06.9: Thyroiditis, unspecified - E05.4: Thyrotoxicosis facticia - E05.5: Thyroid crisis or storm - E04.0: Nontoxic diffuse goitre - E01.0: Iodine-deficiency-related diffuse (endemic) goitre - E01.1: Iodine-deficiency-related multinodular (endemic) goitre - E01.2: Iodine-deficiency-related (endemic) goitre, unspecified - E01.8: Other iodine-deficiency-related thyroid disorders and allied conditions - E07.8: Other specified disorders of thyroid - E07.9: Disorders of thyroid, unspecified - E07.0: Hypersecretion of calcitonin - E07.1: Dyhormonogenetic goitre |
| Study population | Inpatient stays extracted from Hospital administrative database |
| Algorithm | Inpatient stays  extracted from  Hospital administrative database  *NO*  Thyroid pathology code as main diagnosis  EXCLUSION  DENOMINATOR  *NO*  ICD-10 diagnosis code in the numerator list  NUMERATOR |
