# Supplementary material for: Unexpected discrepancies in hospital administrative databases can impact the accuracy of monitoring thyroid surgery outcomes in France
Source: PLoS One. 2018 Dec 6;13(12):e0208416. doi: 10.1371/journal.pone.0208416 (PMC6283582; doi:10.1371/journal.pone.0208416)
Supplement: S3 Fig — (DOCX) [file pone.0208416.s003.docx]

| **S3 Figure.**  Description of the indicator «Postoperative hypoparathyroidism» | |
| --- | --- |
| Definition | The indicator « Postoperative hypoparathyroidism » allows measuring the rate of immediate post-operative hypocalcaemia after a bilateral thyroid surgery, for an institution. |
| Importance | Postoperative hypoparathyroidism  is one of the two potentially preventable adverse events after thyroid surgery. An accurate indicator for this complication could make possible for teams to focus their potential to improve their performance, in order to reduce the occurrence of this complication over time. |
| Type of indicator | Outcome indicator |
| Collection | Continuously collection |
| Data sources | - Hospital admministrative database « Programme de Médicalisation des Systèmes d’Information en Médecine » - Medical record data collection |
| Nature of the indicator | Number of thyroid surgery patients per 100 with postoperative hypoparathyroidism  (rate) |
| Method of calculation | Patients with a ICD-10 diagnosis code of postoperative hypoparathyroidism  = x 100  Inpatient stays with a bilateral thyroid procedure code |
| Numerator | Among the patients in the denominator, patients with one of the following ICD-10 diagnosis codes:   - E89.2: Postprocedural hypoparathyroidism   * ICD-10 codes not retained in the final numerator |
| Denominator | Inclusion criteria:   - Includes inpatients with a thyroid procedure code among: - KCFA010 : Subtotal thyroidectomy, by cervicotomy - KCFA005 : Total thyroidectomy, by cervicotomy - KCFA001 : Totalisation of thyroidectomy, by cervicotomy - KCFA009 : Subtotal thyroidectomy, by cervico-thoracotomy - KCFA007 : Total thyroidectomy, by cervico-thoracotomy - KCFA002 : Total thyroidectomy associated with a laryngeal cartilage resection, by cervicotomy - KCFA003 : Total thyroidectomy associated with a non-interrupted tracheal resection and anastomosis, by cervicotomy - KCFA006 : Total thyroidectomy associated with a interrupted tracheal resection and anastomosis, by cervicotomy   Exclusion criteria:   - Excludes patients without ICD-10 thyroid pathology code as main diagnosis, among: - E04.2: Nontoxic multinodular goitre - E04.1: Nontoxic single thyroid nodule - E04.8: Other specified nontoxic goitre - E04.9: Nontoxic goiter, unspecified - E05.0: Thyrotoxicosis with diffuse goitre - E05.1: Thyrotoxicosis with toxic single thyroid nodule - E05.2: Thyrotoxicosis with toxic multinodular goitre - E05.3: Thyrotoxicosis from ectopic thyroid tissue - E05.8: Other thyrotoxicosis - E05.9: Thyrotoxicosis, unspecified - C73: Malignant neoplasm of thyroid gland - D34: Benign neoplasm of thyroid gland - D44.0: Neoplasm of uncertain or unknown behavior of endocrine gland – thyroid gland - E06.3: Autoimmune thyroiditis - E06.5: Other chronic thyroiditis - E06.9: Thyroiditis, unspecified - E05.4: Thyrotoxicosis facticia - E05.5: Thyroid crisis or storm - E04.0: Nontoxic diffuse goitre - E01.0: Iodine-deficiency-related diffuse (endemic) goitre - E01.1: Iodine-deficiency-related multinodular (endemic) goitre - E01.2: Iodine-deficiency-related (endemic) goitre, unspecified - E01.8: Other iodine-deficiency-related thyroid disorders and allied conditions - E07.8: Other specified disorders of thyroid - E07.9: Disorders of thyroid, unspecified - E07.0: Hypersecretion of calcitonin - E07.1: Dyhormonogenetic goitre - Excludes patients with ICD-10 hyperparathyroidism pathology code as associated diagnosis, among: - E210 : Primary hyperparathyroidism - E211 : Secondary hyperparathyroidism, not elsewhere classified - E212: Other hyperparathyroidism - E213 : Hyperparathyroidism, unspecified - Excludes patients with associated procedure code of parathyroid resection, among : - KDQA001: Parathyroid exploration, by cervicotomy - KDFA002: One parathyroid resection, by cervicotomy |
| Study population | Inpatient stays extracted from Hospital administrative database |
| Algorithm | Inpatient stays  extracted from  Hospital administrative database  *NO*  Thyroid pathology code as main diagnosis  *YES*  Hyperparathyroidism pathology code  EXCLUSION  *YES*  Procedure code of parathyroid resection  DENOMINATOR  *NO*  ICD-10 diagnosis code in the numerator list  NUMERATOR |
